# The stratum corneum comprises three layers with distinct metal-ion barrier properties

**DOI:** 10.1038/srep01731

**Published:** 2013-04-25

**Authors:** Akiharu Kubo, Itsuko Ishizaki, Akiko Kubo, Hiroshi Kawasaki, Keisuke Nagao, Yoshiharu Ohashi, Masayuki Amagai

**Affiliations:** 1 Department of Dermatology, Keio University School of Medicine, Tokyo 160-8582, Japan; 2 Center for Integrated Medical Research, Keio University School of Medicine, Tokyo 160-8582, Japan; 3 ULVAC-PHI INC., Chigasaki 253-0084, Japan; 4 Department of Biochemistry, Keio University School of Medicine, Tokyo 160-8582, Japan

## Abstract

The stratum corneum (SC), the outermost barrier of mammalian bodies, consists of layers of cornified keratinocytes with intercellular spaces sealed with lipids. The insolubility of the SC has hampered in-depth analysis, and the SC has been considered a homogeneous barrier. Here, we applied time-of-flight secondary ion mass spectrometry to demonstrate that the SC consists of three layers with distinct properties. Arginine, a major component of filaggrin-derived natural moisturizing factors, was concentrated in the middle layer, suggesting that this layer functions in skin hydration. Topical application of metal ions revealed that the outer layer allowed their passive influx and efflux, while the middle and lower layers exhibited distinct barrier properties, depending on the metal tested. Notably, filaggrin deficiency abrogated the lower layer barrier, allowing specific metal ions to permeate viable layers. These findings elucidate the multi-layered barrier function of the SC and its defects in filaggrin-deficient atopic disease patients.

The stratum corneum (SC), the horny layer of the mammalian epidermis, directly faces the external environment and protects the inner viable layers from desiccation and foreign insult. Recent findings have shown that disruption of epidermal barrier systems, for example, filaggrin deficiency, is involved in the pathogenesis of atopic diseases via augmented percutaneous sensitization with allergens that penetrate the body through the abrogated barrier of the SC^1^,^2^,^3^,^4^. Contact sensitization against small metal ions such as nickel has been associated with filaggrin deficiency, suggesting that filaggrin deficiency affects the barrier properties of the SC even against small metal ions^5^,^6^,^7^.

The SC consists of layers of cornified keratinocytes (corneocytes) attached to each other by corneodesmosomes, with intercellular spaces sealed with lipids. The intracellular space of corneocytes is filled with keratin filaments, filaggrin, and their degradation products^2^,^8^. Each corneocyte is encased in a “cornified envelope,” an insoluble amalgam of proteins highly cross-linked by transglutaminases, the surface of which is tightly bound to intercellular lipids, providing a barrier against the passage of water and water-soluble substances^9^,^10^.

The SC functions as an outside-in barrier against foreign insults as well as an inside-out barrier to keep skin hydrated. The insolubility of the SC and its densely packed and heavily cross-linked proteins has hampered the detailed analysis of its barrier nature by conventional imaging techniques. To overcome these difficulties, we applied imaging mass spectrometry (MS) in this study to analyze the SC. Imaging MS of biological samples allows visualization of the spatial distribution of molecules on a sample, typically a thin tissue section, by ionizing molecules from each X-Y point, then analyzing the ionized molecules and molecular ion fragments by MS and identifying them by their mass-to-charge ratio (*m/z*).

The SC has been considered a homogeneous barrier, and whether it consists of functionally distinct layers is unknown. Previous studies suggested that topically applied small molecules distribute more within upper layers than in lower ones; however, these results have been interpreted as a simple diffusion gradient of applied molecules within the SC^11^,^12^,^13^. Here, we applied imaging MS technology using time-of-flight secondary ion MS (TOF-SIMS) to analyze the SC. In contrast to previous studies using MS^14^,^15^, our improved sample preparation method and TOF-SIMS enabled us to visualize the spatial distribution of natural substances and externally applied molecules simultaneously in submicron spatial resolution. This approach revealed that the SC consists of three sharply demarcated layers with distinct barrier properties.

## Results

### TOF-SIMS imaging of freeze-dried murine tissue sections

**Figure 1:**
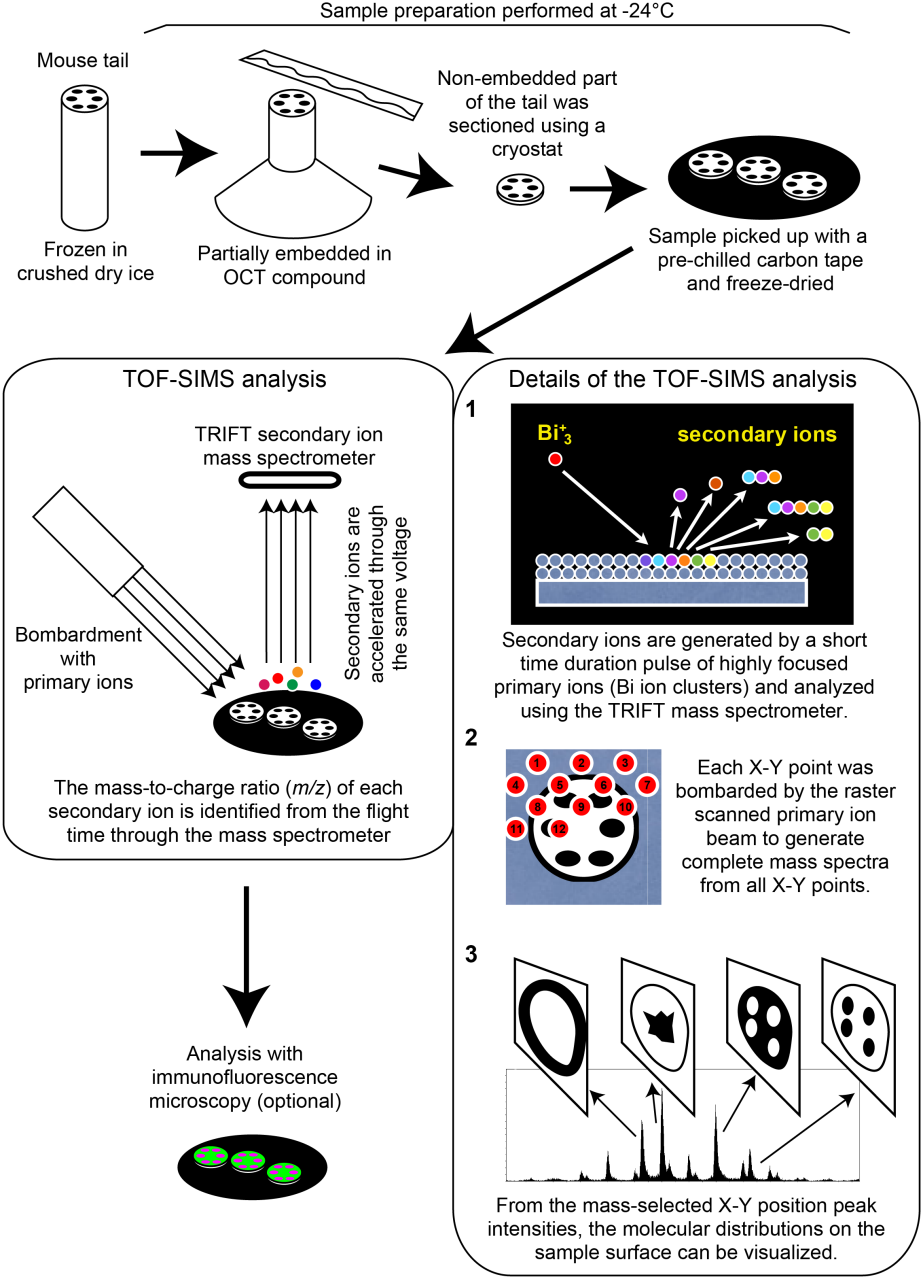
Diagram of the TOF-SIMS analysis work flow. A schematic illustration of the sample preparation, TOF-SIMS analysis, TOF-SIMS data analysis and optional sequential analysis in immunofluorescence microscopy of the SC cross-section samples.

**Figure 2:**
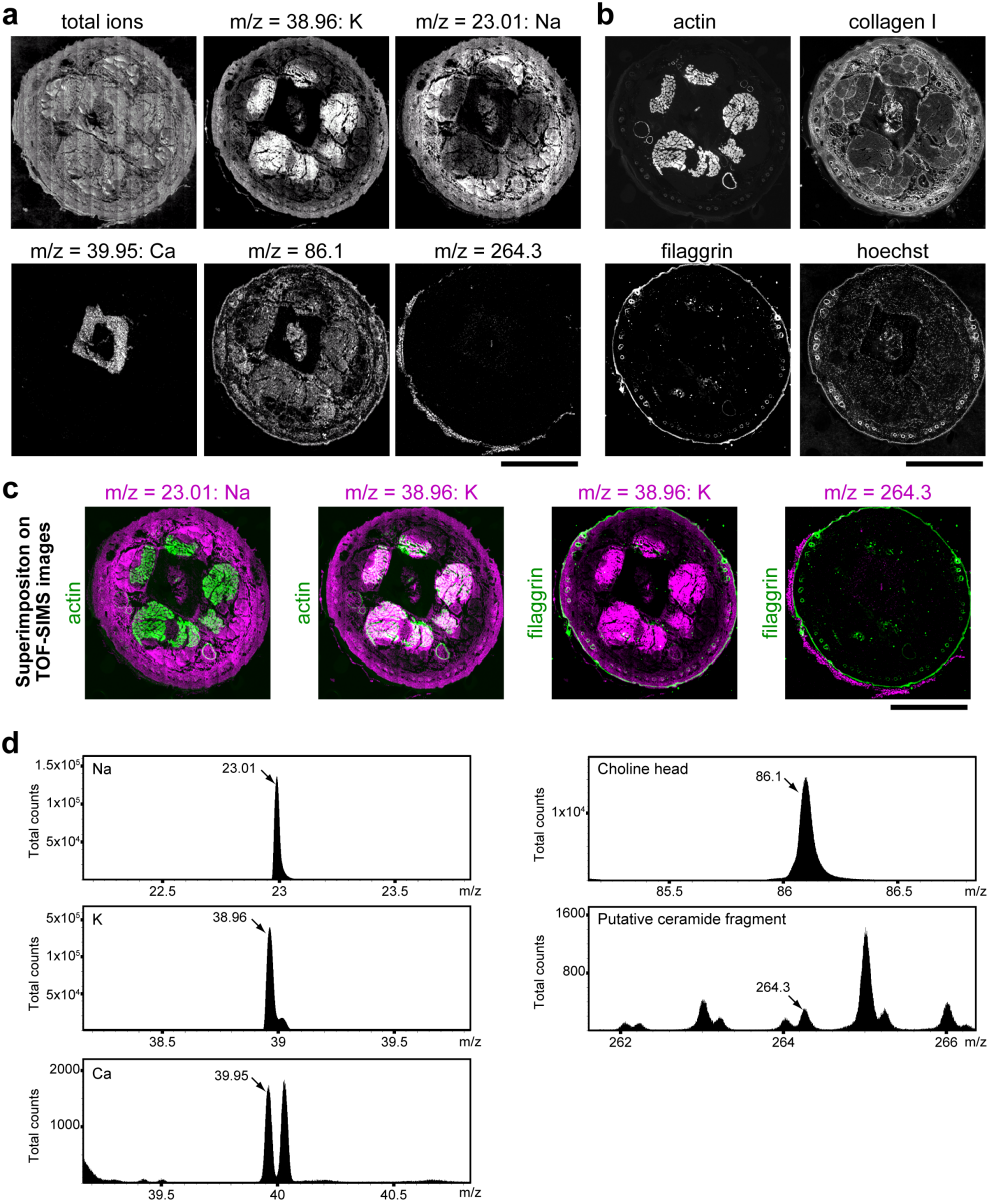
Sequential visualization of mouse tail sections by TOF-SIMS and immunofluorescence. (a) Representative positive-ion micrographs of a flash-frozen, freeze-dried mouse tail section, showing the spatial distribution of signals of the indicated m/z peaks, which are defined in (d). (b) Immunofluorescence images of the same mouse tail section analyzed in (a). Actin staining shows six muscles of the mouse tail, and filaggrin staining shows the granular layer of the epidermis. (c) *In silico* superimposition of the TOF-SIMS images of the indicated m/z peaks (purple) on the immunofluorescence images of the indicated proteins (green). (d) Positive-mode TOF-SIMS mass spectra in the indicated m/z range from a mouse tail section. The spatial distribution of the molecules of the indicated peaks (arrows) is presented in (a). Each image is representative of three mice, for each of which two tail sections were investigated. Scale bars, 1 mm (a–c).

We prepared sections of flash-frozen mouse tails and freeze-dried the sections without sample thawing to minimize delocalization of non-fixable molecules (see Methods). The sections were directly analyzed by TOF-SIMS to visualize the distribution of natural substances without applying a staining procedure and were subsequently processed for immunofluorescence (Fig. 1). The obtained fluorescent images were superimposed on the corresponding TOF-SIMS images *in silico*. Muscles were visualized as actin^high^ by immunofluorescence and potassium (K)^high^ sodium (Na)^low^ in TOF-SIMS, and connective tissues were visualized as collagen I^high^ in immunofluorescence and K^low^ Na^high^ in TOF-SIMS (Fig. 2a–c), suggesting that cell-rich areas are K^high^ Na^low^ and that matrix-rich areas are K^low^ Na^high^, probably due to the exchange of Na and K ions on the cell surface^16^,^17^. Bone was visualized as calcium (Ca)^high^ areas (Fig. 2a). Along the outside of the filaggrin-positive granular layer^18^, a positive-ion peak of putative ceramide fragments (*m/z* = 264.3)^19^ was exclusively seen in the SC (Fig. 2a–c). Positive-mode TOF-SIMS mass spectra of each ion are presented in Fig. 2d.

### Identification of the SC in skin sections by TOF-SIMS

**Figure 3:**
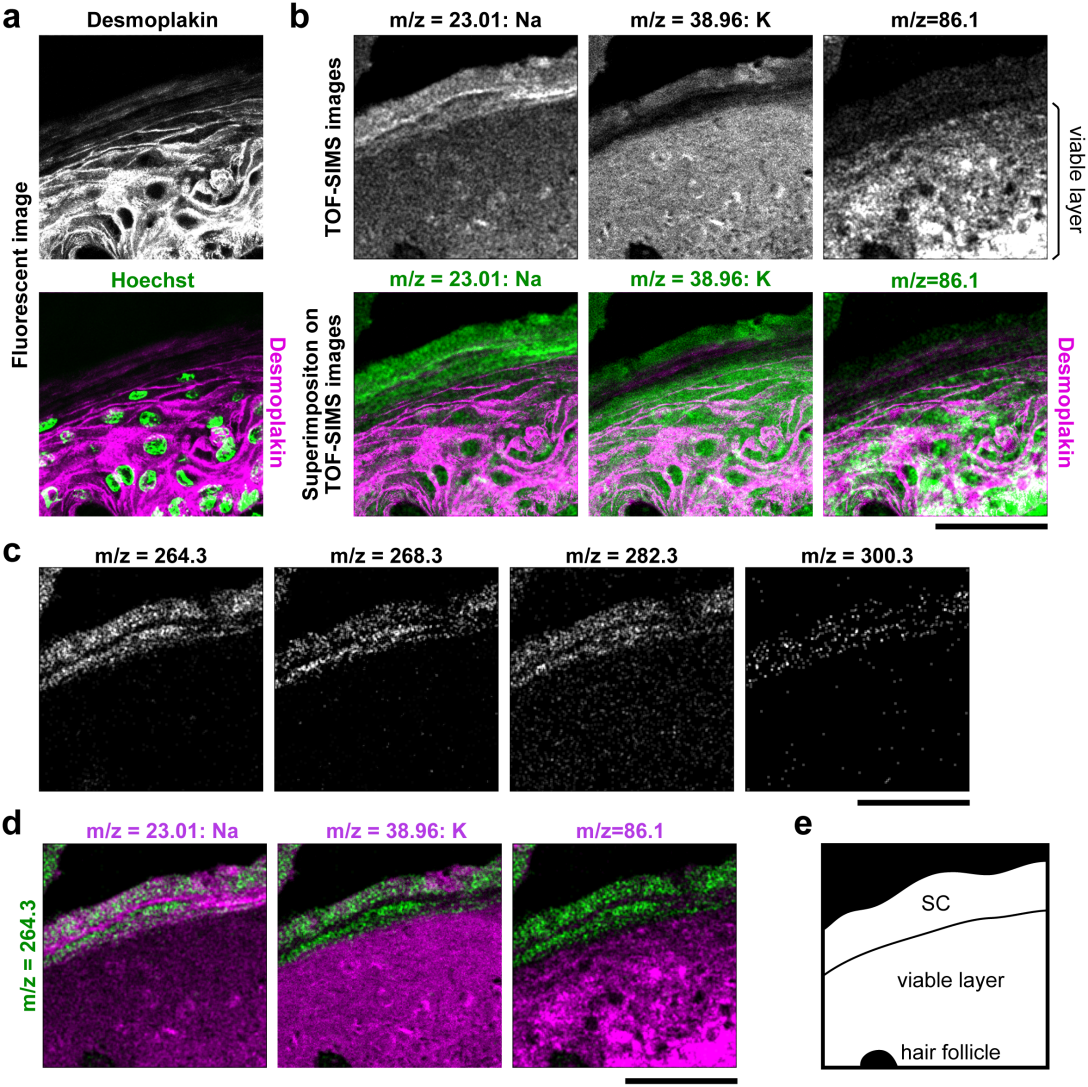
Sequential visualization of mouse skin sections by TOF-SIMS and immunofluorescence. (a–d) Representative immunofluorescent images and TOF-SIMS high-resolution positive-ion micrographs of a mouse skin section. The superimposition procedure is shown in Supplementary Fig. 1. (a) Immunofluorescent desmoplakin and Hoechst staining of mouse skin. (b) TOF-SIMS high-resolution positive-ion micrographs of the same area shown in (a). TOF-SIMS images show the distribution of signals from the indicated *m/z* peaks defined in Supplementary Fig. 2 (upper panels). The immunofluorescent image of desmoplakin (purple) was superimposed on the positive-ion micrographs (green; lower panels). (c) Spatial distribution of putative ceramide fragments (*m/z* = 264.3, 268.3, 282.3, and 300.3) in the same area shown in (a). Each *m/z* peak is defined in Supplementary Fig. 3. (d) TOF-SIMS image of the *m/z* = 264.3 peak (green) is co-visualized with the other molecules (purple) presented in (b, upper panels). Scale bars, 50 μm. (e) Schematic representation of the analyzed area. Each image is representative of three mice, for each of which two tail sections were investigated.

Next, we analyzed 100 μm square areas of skin in mouse tail sections by TOF-SIMS followed by immunofluorescence imaging. In the immunofluorescent images, the viable keratinocyte layer and the SC were clearly distinguished by the staining of desmoplakin, loricrin, and nuclei (Supplementary Fig. 1a). We superimposed the TOF-SIMS images on the corresponding immunofluorescent images *in silico* using hair follicles and debris as reference points and determined the border between the SC and viable cell layer (Fig. 3 and Supplementary Fig. 1b). There were some methodological limitations to superimposing images gained by two different methods (see Methods). Nonetheless, the border appeared clear on the image of Na/K distribution, where the Na^low^ K^high^ viable layer transitioned to Na^high^ K^low^ in the SC (Fig. 3a, b and Supplementary Fig. 2), probably due to the termination of ATP-dependent Na–K exchange on cell membranes undergoing cornification. In the upper layer of the SC, the concentration of K ions became high again (Fig. 3b). This K likely originated from the external environment, as described below.

Positive fragment ions of choline (*m/z* = 86.1), which is derived from intracellular membranes^17^, were highly detected in the viable layer but not in the SC, demonstrating the total disappearance of cytoplasmic organelles under cornification^20^ (Fig. 3b and Supplementary Fig. 2). Several peaks within the *m/z* range of 260–300 were specifically detected in the SC (Fig. 3c, d). As one peak (*m/z* = 264.3) has been reported as resulting from a fragment of ceramide^19^, we analyzed purified ceramide 6, one of the SC ceramides, by TOF-SIMS. Four major peaks of *m/z* = 264.3, 268.3, 282.3, and 300.3 were detected, all of which were specifically detected in the SC (Fig. 3c, d and Supplementary Fig. 3), indicating that TOF-SIMS successfully detected a significant portion of SC ceramide. Integrating these results comprehensively, we defined the SC as Na^high^ ceramide^high^ choline^low^ and the viable cell layer as Na^low^ ceramide^low^ choline^high^.

### Three layers in the SC with an arginine-rich layer in the middle

Filaggrin deficiency is the major predisposing factor for atopic dermatitis. Mature filaggrin proteins are produced from protease-dependent processing of profilaggrin under cornification and align keratin filaments into highly ordered and condensed arrays in the lower layers of the SC^2^,^21^,^22^,^23^. Filaggrin is degraded into so-called natural moisturizing factors (NMFs) within corneocytes and functions in skin hydration.

**Figure 4:**
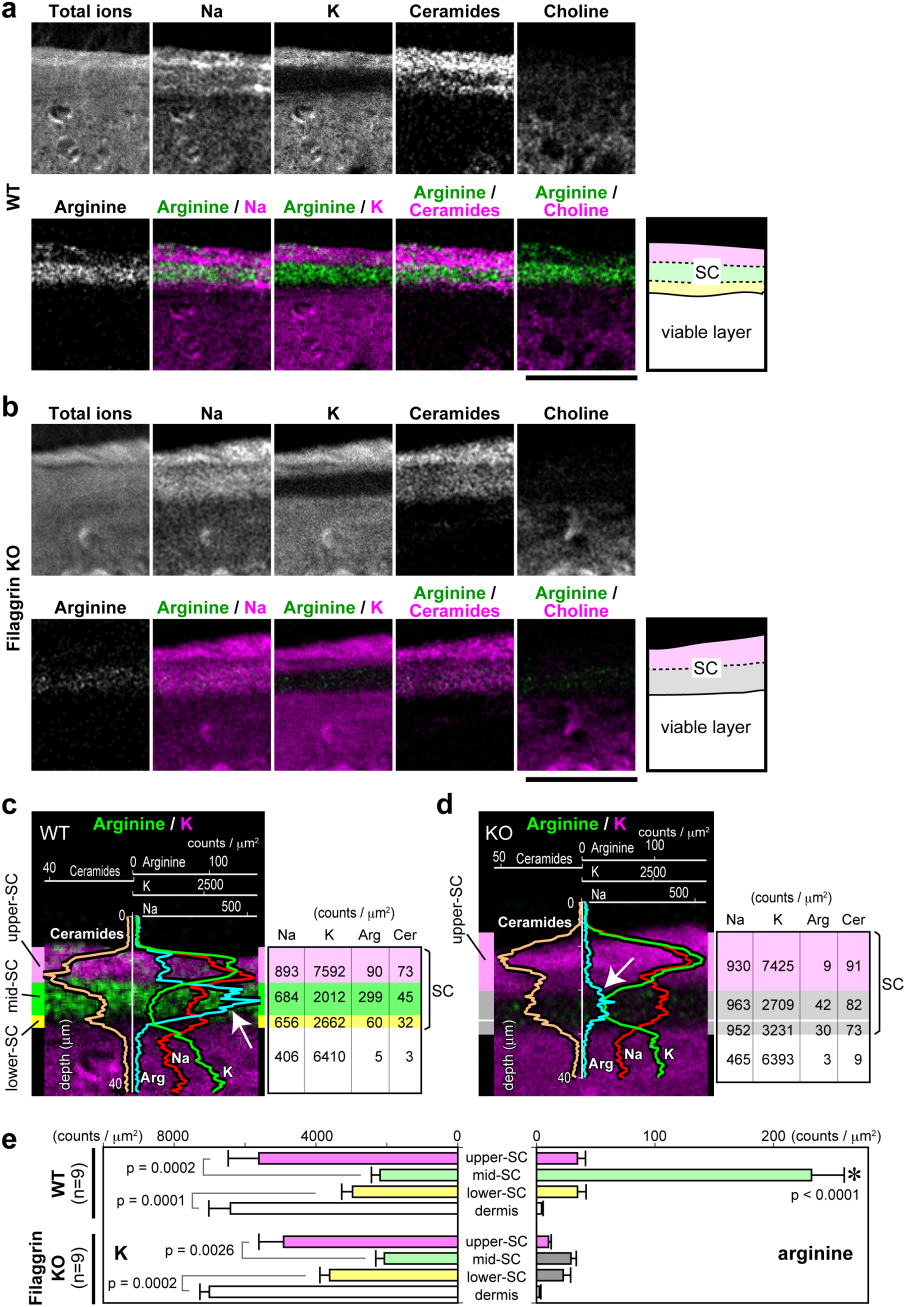
Detection of filaggrin-derived arginine reveals three distinct layers of the SC. (a, b) Representative positive-ion micrographs of skin sections from wild-type (WT) (a) and filaggrin-knockout (KO) mice (b). Schematic representation of the analyzed area is shown on the right. The positive-ion micrograph of arginine (lower left) is co-visualized in green with other molecules in purple (lower panels). The ion peaks of arginine from WT and filaggrin-KO mice are defined in Supplementary Fig. 4. Scale bars, 50 μm. (c, d) The line scan data of each ion signal of the represented areas in a and b, respectively, overlaid on the images of arginine/K. The average signal count of each area is shown to the right. The SC in WT skin is striped with the K^high^ arginine^low^ upper SC (purple), K^low^ arginine^high^ mid SC (green), and K^low^ arginine^low^ lower SC (yellow) layers. In filaggrin-KO mice, the mid and lower SC layers were indistinguishable and are both illustrated in gray. Each image in a–d is representative of three mice, for each of which at least three tail sections were investigated. (e) The average arginine and K signal counts of each area for nine section views from three mice. When the average K signal counts were compared between neighboring layers, significant differences were detected between the upper SC (WT; 5609 ± 866.1 (*mean* ± *S.E.M.*), KO; 4914 ± 695.4) and the mid SC (WT; 2197 ± 246.1, KO; 2070 ± 241.5) or between the lower SC (WT; 2967 ± 308.4, KO; 2615 ± 273.0) and the dermis (WT; 6412 ± 610.0, KO; 7006 ± 268.0). No significant difference was detected between each corresponding layer of the WT and KO skin. *The average arginine signal counts for the mid SC of WT mice (232.1 ± 27.7) was significantly higher (*P* < 0.0001) than those of other areas (WT upper SC, 34.5 ± 6.8; WT lower SC, 34.6 ± 7.2; WT dermis, 4.2 ± 1.3; KO upper SC, 10.1 ± 2.3; KO mid SC, 29.1 ± 4.4; KO lower SC, 22.5 ± 6.2; and KO dermis, 2.4 ± 1.3).

First, we sought to determine the spatial distribution of NMFs within the SC by comparing the ion peaks detected in the SC from wild-type (WT) versus filaggrin-knockout (KO) mice. We identified an ion peak of *m/z* = 175.1, which was specifically reduced in filaggrin-KO SC (Fig. 4a–d and Supplementary Fig. 4). The *m/z* value highly suggests that this peak is due to a free arginine molecule, one of the major NMFs reduced in filaggrin-KO SC^18^, which was confirmed by TOF-SIMS analysis of purified arginine (Supplementary Fig. 4).

We noticed that the spatial distribution of K and arginine reproducibly stratifies the SC into three layers (Fig. 4a, c). Most of the arginine was specifically concentrated in the middle layer of the SC in WT mice (Fig. 4e), which markedly decreased in filaggrin-KO mice, suggesting that this layer functions in skin hydration. The border of the lower arginine^low^ layer and the arginine^high^ middle layer appeared sharp in the WT SC (Fig. 4a, c), indicating that the production of arginine, and probably other NMFs, from filaggrin is tightly controlled. In the K^high^ upper layer of the WT SC, the amount of arginine markedly decreased, suggesting its further modification (Fig. 4a, c, e). Thus, we divide the SC into three layers according to the distribution of K and arginine: an upper K^high^ arginine^low^ layer (upper SC), a middle K^low^ arginine^high^ layer (mid SC), and a thin innermost K^low^ arginine^low^ layer (lower SC), as shown in Fig. 4a, c and e. In filaggrin-KO mice, weak arginine signals were still detected in the lower K^low^ layer, suggesting the existence of minor NMF resources other than filaggrin (Fig. 4b, d, e). When the lower K^low^ layer was provisionally divided into two sub-layers (putative mid SC and lower SC), no significant difference between the average arginine signal counts of the two layers was detected (Fig. 4d, e).

### The upper SC allows the passive influx and efflux of exogenous ions

**Figure 5:**
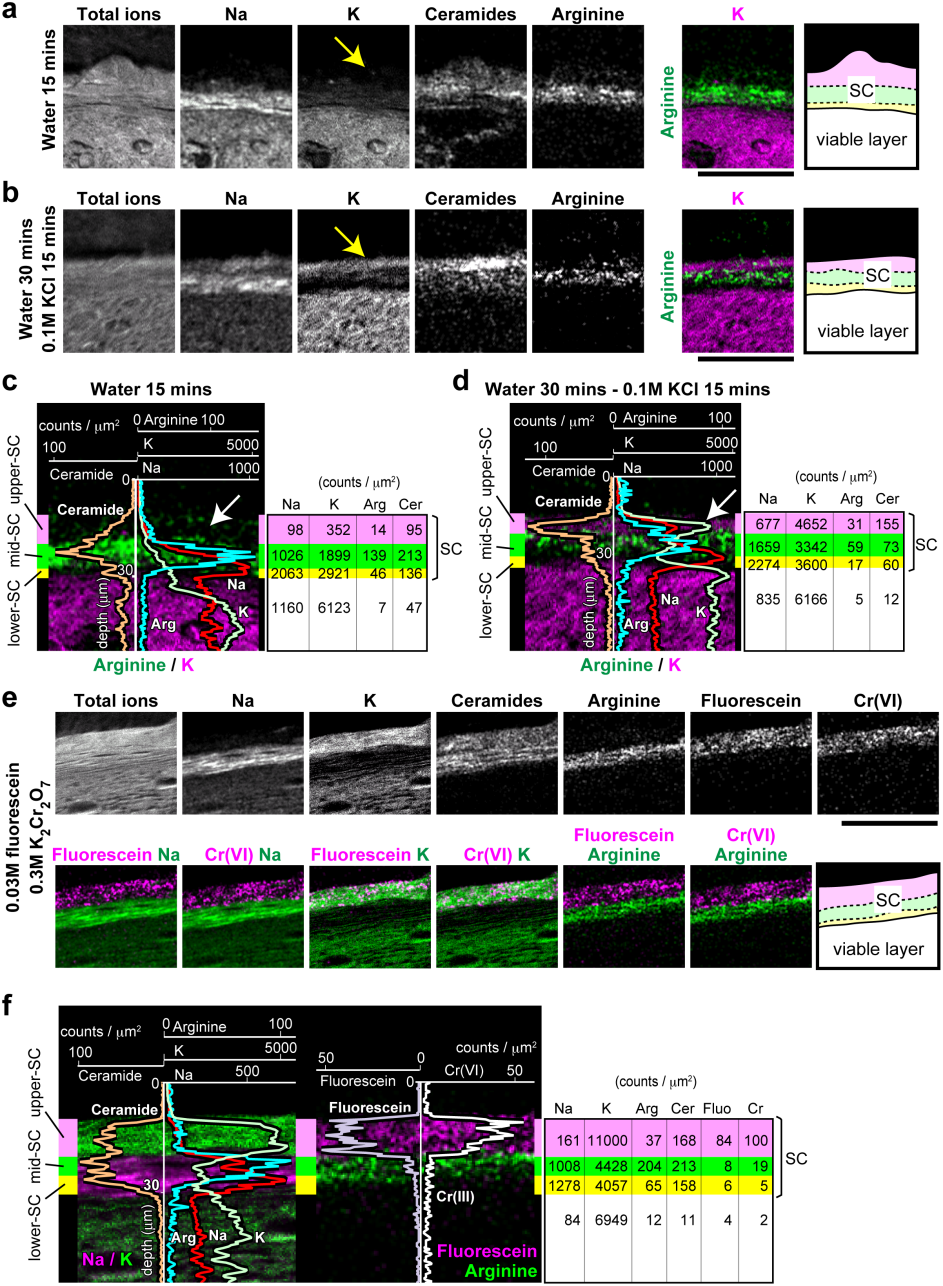
Infiltration of externally applied molecules into the upper SC, but not the mid SC. (a, b) Representative positive-ion micrographs of skin sections of mouse tails after soaking in water for 15 min (a) and after 30 min followed by soaking in 0.1 M KCl solution for 15 min (b). Schematic representation of the analyzed area is shown on the right. Scale bars, 50 μm. (c, d) Line scan data for each ion signal for the areas in (a) and (b), respectively, overlaid on the image of arginine/K. The three distinct layers of the SC are illustrated by three colors (purple, green, and yellow) from outside inward. The average signal count for each area is shown on the right. Arrows indicate the washout and reappearance of K in the upper SC. (e) Representative positive-ion micrographs of skin sections of mouse tails after soaking in 0.03 M fluorescein/0.3 M K_2_Cr_2_O_7_ water solution for 45 min. The *m/z* peaks of Cr and fluorescein are defined in Supplementary Figs 6 and 7, respectively. Schematic representation of the analyzed area is shown on the right. Scale bar, 50 μm. (f) Line scan data for Na, K, arginine, ceramide, fluorescein, and Cr(VI) from the area in (e) overlaid on the images of Na/K and fluorescein/arginine. The three distinct layers of the SC are illustrated in three colors (purple, green, and yellow). The average signal count for each area is shown on the right. Images in a–f are representative of three mice, for each of which at least three tail sections were investigated.

To investigate the properties of these three distinct SC layers with regard to outside-in barrier function, we performed soaking assays. First, tails of live mice were soaked in water for 15 min and processed for TOF-SIMS imaging. K in the upper SC markedly reduced, resulting in the entire SC becoming equally K-negative (Fig. 5a, c). Na also decreased in the upper SC, but not in the mid or lower SC layers. The distribution of ceramide and arginine appeared unchanged (Fig. 5a, c). When the water-soaked tails were then subjected to soaking in 0.1 M KCl solution for 15 min, K reappeared in the upper SC (Fig. 5b, d).

To further investigate the permeability of the upper SC, we soaked mouse tails in solutions containing several different metal ions. The metal ions used were chosen based on their ability to be detectable by TOF-SIMS and distinguishable from other ion peaks of naturally existing molecules in the skin; thus, we selected chromium (Cr) ions. We soaked the tails in 0.3 M K_2_Cr_2_O_7_ water solution to investigate the influx of hexavalent Cr (Cr(VI)). Cr(VI) ions were detected exclusively in the upper SC after soaking for 15, 45, and 90 min (Supplementary Fig. 5 and data not shown; Cr ion peaks are shown in Supplementary Fig. 6). The distribution of K, ceramide, and arginine appeared unchanged, while Na in the upper SC, but not in the mid SC, decreased (Supplementary Fig. 5). The disappearance of intra-corneocyte Na from the upper SC strongly suggests that the solution directly penetrated corneocytes and washed out the intra-corneocyte Na. Thus, the upper SC is suggested to be a specific layer that allows the passive influx and efflux of exogenous ions.

### The mid SC works as a first line of defense

These soaking experiments highlighted the mid SC as a barrier against metal ions. To confirm the relevance of TOF-SIMS imaging in the soaking assay, we soaked mouse tails in 0.03 M fluorescein/0.3 M K_2_Cr_2_O_7_ water solutions to compare the distribution of fluorescein as detected by TOF-SIMS versus fluorescence microscopy. A specific peak of fluorescein (*m/z* = 333.1) was detected from the desiccated fluorescein solution and from the SC of soaked tails, but not from control tails (Supplementary Fig. 7). The fluorescein soaked into the upper SC but not into the mid SC, as did K and Cr(VI) according to TOF-SIMS images (Fig. 5e, f). Sequential analysis of the same specimen by fluorescence microscopy confirmed the spatial distribution of fluorescein observed by TOF-SIMS (Supplementary Fig. 8). These observations indicated that the mid SC functions as a barrier against K and Cr(VI) ions as well as against fluorescein.

### The lower SC works as a second line of defense

**Figure 6:**
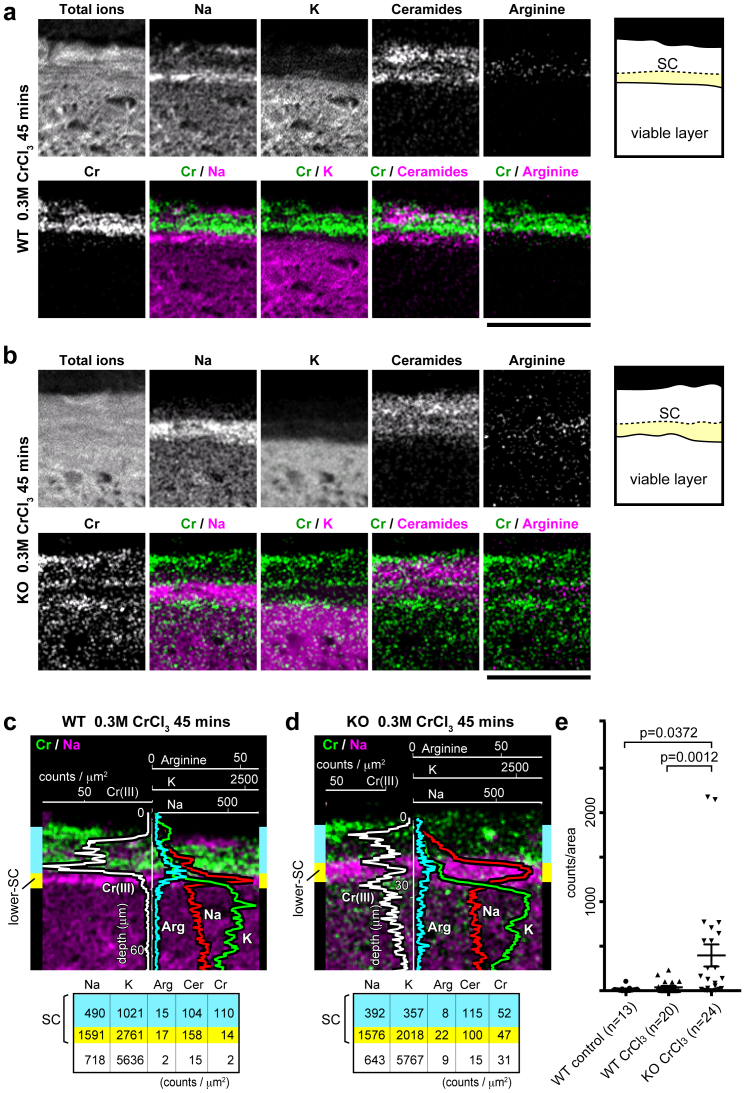
Distinct barrier function of the mid SC and the lower SC. (a, b) Representative positive-ion micrographs of skin sections from a wild-type (WT) mouse (a) and a filaggrin-knockout (KO) mouse (b) after soaking in 0.3 M CrCl_3_ solution for 45 min. Schematic representations of the analyzed areas are shown on the right. Scale bars, 50 μm. (c, d) Line scan data for Na, K, arginine, and Cr(III) from the areas shown in (a) and (b), respectively, overlaid on the images of Cr/Na. The upper and mid SC layers were indistinguishable and are both illustrated in blue, while the lower SC is illustrated in yellow. The average signal count for each area is shown at the bottom. Images presented in a–d are representative of three mice, for each of which at least three sections were investigated. (e) Total Cr(III) signals detected from 50 × 20 μm square areas of viable cell layers, located just beneath the SC, were compared between non-application control WT mice (13 views of three tail sections from three mice), CrCl_3_-applied WT mice (20 views of six tail sections from three mice), and CrCl_3_-applied filaggrin-KO mice (24 views of six tail sections from three mice). Statistical analysis revealed a significant increase in Cr(III) in the viable layer of the epidermis in filaggrin-KO mice (399.4 ± 125 count/area (*mean ± S.E.M.*)) compared with WT mice after topical application of CrCl_3_ (38.9 ± 15.4; *P* = 0.0012) or non-application control WT mice (17.4 ± 7.8; *P* = 0.0372).

Next, we investigated the difference between the mid SC and lower SC with regard to barrier function. While the mid SC blocked K and Cr(VI) ions, we found that trivalent Cr (Cr(III)) ions soaked into deeper layers of the SC when tails of live mice were soaked in 0.3 M CrCl_3_ solution for 45 min (Fig. 6a, c). In Cr(III)-diffused areas, the arginine signals of the mid SC became mostly undetectable, and the Na signals of the upper and mid SC layers markedly decreased. Beneath the Cr(III)-diffused area, a thin SC layer of Na^high ^Cr(III)^negative ^remained, suggesting that Cr(III) soaked into the corneocytes of the upper and mid SC but not into the lower SC (Fig. 6a, c). The blocking of Cr(III) influx into the lower SC remained unchanged when the concentration of CrCl_3_ was elevated to 1 M or the soaking time was extended to 90 min (data not shown). These results indicate that the lower SC functions as a barrier that differs from that of the mid SC.

### Filaggrin deficiency affects the lower SC barrier against Cr(III)

**Figure 7:**
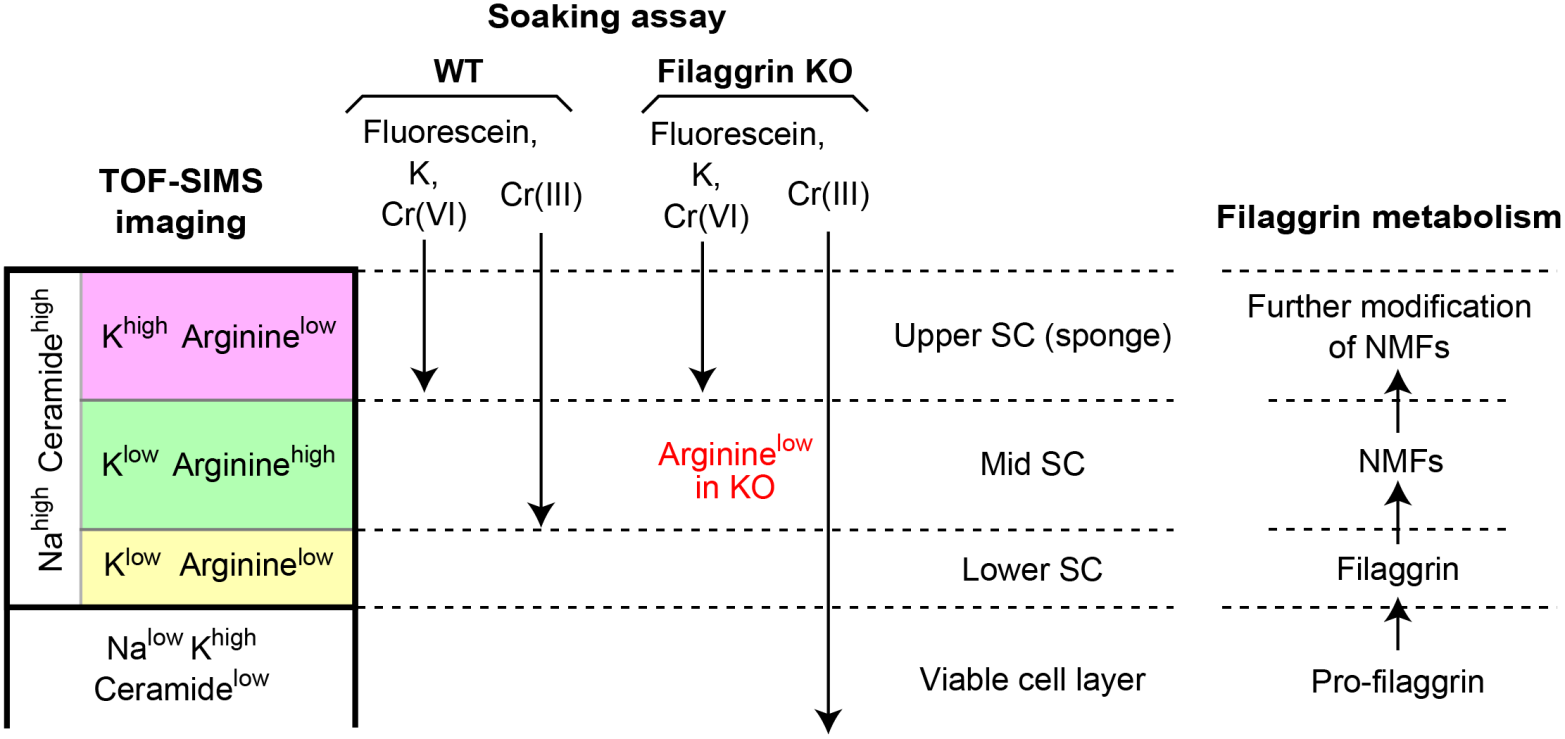
Schematic drawing of the three layers of the SC. Summary of the distribution of internal molecules and externally applied molecules in the three SC layers as well as the viable cell layer. Putative correspondence of the SC layers with filaggrin metabolism is shown on the right.

To investigate whether filaggrin deficiency affected either of the two distinct barriers observed here, soaking assays were performed in filaggrin-KO mice. No apparent difference was observed in the barrier function of the mid SC against the influx of fluorescein, K, or Cr(VI) in filaggrin-KO versus WT mice (data not shown). These results are consistent with our previous observations that calcein water solution only penetrated the upper layer of the SC both in WT and filaggrin-KO SC^18^. In contrast, Cr(III) was observed to focally permeate into the viable layer of the epidermis in spots, in filaggrin-KO mice (Fig. 6b, d). Statistical analysis confirmed the significant increase of Cr(III) detected in the viable layer of filaggrin-KO mice compared with WT or control mice (Fig. 6e). Note that even in the areas where Cr(III) soaked into the viable layers, Na signals from the lower SC mostly remained (Fig. 6b), suggesting that the intra-corneocyte space of the lower SC corneocytes is not directly saturated with Cr(III) ions, although the integrity of the lower SC, consisting of the corneocyte and inter-corneocyte lipids, was affected. Therefore, among the three SC layers with functionally distinct properties, filaggrin deficiency appeared to have no impact on barrier function of the mid SC, where arginine and probably other NMFs markedly decreased, but specifically affected the integrity of the lower SC layer (Fig. 7).

## Discussion

In this study, we applied TOF-SIMS to investigate the function of the SC. TOF-SIMS imaging and matrix-assisted laser desorption ionization (MALDI)-MS imaging are similar techniques that have several advantages, including the lack of the need for fixation or chemical labeling, which avoids delocalization of unfixable molecules, and the ability to simultaneously image a variety of biological compounds in a single run^24^,^25^. The lateral resolution of MALDI-MS imaging depends on the laser spot size, which is greater than 10 μm^26^, and the use of a matrix further lowers the lateral resolution. In contrast, the lateral resolution of TOF-SIMS is hundreds of nanometers^25^, which allowed us to use TOF-SIMS for the SC analysis. The imaging capabilities of TOF-SIMS of biological samples are generally limited to low-mass ions (<2,000 daltons), and the variety of identifiable molecules is much lower than that for MALDI-MS. This has limited the biological applications of TOF-SIMS imaging^24^,^25^. In this study, we used bismuth cluster ions as a primary ion source^25^, which enabled us to detect fragments with relatively high masses (*i*.*e*., arginine, ceramide fragments, and fluorescein) for analysis of the SC barrier. MALDI-MS is not suitable for the detection of amino acids or low-mass ions such as Cr observed in this study. Furthermore, TOF-SIMS only sputters the surface molecules (to about 1 nm in depth) of samples in contrast to MALDI-MS, which samples a depth greater than 1 μm, allowing analysis of the same location on a sample sequentially by histological examination after the MS analysis.

Using TOF-SIMS, we found that the SC consists of three distinct layers that likely correspond to the metabolic processing of filaggrin. The arginine^low^ lower SC, which functions as an outside-in barrier, is consistent with the innermost SC harboring interlaced keratin patterns bundled by mature filaggrin that physically stabilize the corneocyte keratin framework^2^,^20^,^21^,^27^, which is lost under filaggrin-deficient conditions^18^. Increased susceptibility to mechanical stress may induce focal barrier breakage in the lower SC of filaggrin-KO skin. In the analysis of filaggrin-KO mice, we identified premature detachment of corneocytes rather than direct destruction of corneocytes^18^. Together with the observation that intra-corneocyte Na is mostly preserved in the lower SC, even in the Cr(III)-permeated area, the barrier integrity of the inter-corneocyte space rather than the corneocyte itself might be affected in the filaggrin-KO lower SC (*i*.*e*., easy deformation of filaggrin-deficient corneocytes subjected to mechanical stress or weakening of inter-corneocyte corneodesmosome junctions breaks the barrier of the inter-corneocyte lipid lamellar structure). Although remnants of tight junction proteins were detected in the SC by immunoelectron microscopy^28^,^29^, their contribution to the water-repellant barrier of the SC remains in doubt because claudin-1-KO mice showed no apparent barrier defect in the para-corneocyte pathway of the SC^30^.

The mid SC, which also functions as an outside-in barrier, is rich in arginine and probably other NMFs produced from filaggrin, suggesting that it acts as a hydration layer^31^. The arginine level of the mid SC markedly decreased in filaggrin-KO mice, which made it difficult to distinguish the mid and the lower SC by arginine imaging in KO mice. Small amounts of arginine were still detected in the SC of filaggrin-KO mice, and likely originated from other minor sources of NMFs, such as filaggrin 2^32^,^33^,^34^. The SC hydration of filaggrin-KO mice showed no significant decrease at 22–26°C and 40–60% humidity^18^. Further investigations including estimation of environmental effects, e.g., low humidity, on SC hydration, are needed to explore the contribution of NMFs to SC hydration.

The upper SC works like a “sponge,” where solutes (*e*.*g*., ions contained in sweat or antimicrobial molecules) flow in and some are retained. The low arginine levels in the upper SC are probably due to further metabolic modification, i.e., citrullination^22^, or direct loss to the external environment, and the K in the upper SC likely originates from the external environment, e.g., smears of urine. As the intra-corneocyte Na was washed out from the upper SC in soaking experiments, externally applied molecules would directly infiltrate the corneocytes of the upper SC. A candidate route for this trans-corneocyte pathway is permeation through degenerated corneodesmosomes, as has been demonstrated for mercury chloride permeation^35^. This pathway will likely facilitate an understanding of the trans-corneocyte infiltration of aqueous solutions specifically observed in the upper SC because corneodesmosomes are only limitedly degraded in the upper layers of the SC^36^,^37^,^38^,^39^.

Small metal ions have been shown to penetrate the skin^11^,^12^,^40^,^41^,^42^,^43^,^44^,^45^,^46^. Our study is the first to show that fine structures within the SC absorb or repel small metal ions. Cr(III), but not Cr(VI), has binding activity with various molecules, including amino acids and proteins^47^, suggesting that this highly reactive property of Cr(III) facilitates deeper infiltration. Nickel allergy may be associated with filaggrin mutations due to increased risk of the metal's penetration through the SC^5^,^6^,^7^. Although we failed to visualize the infiltration of nickel ions because of the presence of molecules with the same *m/z* value within mouse skin (data not shown), our data suggest that the SC barrier could be weakened against penetration of particular small metal ions, e.g. Cr(III), by filaggrin deficiency.

We do not know which kinds of molecules are able to penetrate the epidermis and enhance percutaneous cellular and humoral immune responses in filaggrin-deficient people, which must be an early and important step in the pathogenesis of atopic dermatitis^1^,^3^. Further evaluation of the molecular aspects of the three SC layers, as well as assessment of the permeation of various molecules including haptens, will provide us with a better understanding of the SC functions and corresponding deficiencies that contribute to the development of allergic diseases.

## Methods

### Animals

Female C57B6/J mice and filaggrin-KO mice^18^ (6–8 days old) were used in all experiments. Soaking assays were performed with anesthetized mice by soaking their tails in water or water solutions of 0.1 M KCl, 0.03 M fluorescein, 0.3 M K_2_Cr(VI)_2_O_7_, or 0.3 M, 0.6 M, or 1 M Cr(III)Cl_3_ for the indicated times. All animal protocols were approved by the animal ethics review board of Keio University and conformed to National Institutes of Health guidelines.

### TOF-SIMS sample preparation

Solutions of ceramide 6, arginine, K_2_Cr_2_O_7_, CrCl_3_, and fluorescein were desiccated on a silicon wafer for the TOF-SIMS analysis. For the mouse tissue analysis, the tails of 6–8-day-old mice were rapidly frozen in crushed dry ice and cut into 1-cm-long sections at –24°C. A small mound of OCT compound (Sakura Finetek, Tokyo, Japan) was mounted on the prechilled cryostat base, and the tail was stuck into the mound. Thus, we omitted sample embedding for sectioning. The upper part of the tail, the lower part of which was fixed by the mound, was sectioned into 12-μm-thick pieces by cryostat (Leica Microsystems, Wetzlar, Germany). All procedures were done at −24°C. To avoid thawing and delocalization of molecules, the sections were directly pasted on prechilled electrically conductive carbon tapes (Nisshin EM, Tokyo, Japan) on glass coverslips at −24°C, freeze-dried for more than 10 h within the cryostat, and preserved at −80°C in airtight plastic tubes in the presence of silica gel until analysis by TOF-SIMS. The sample preparation and analysis procedure is schematically illustrated in Fig. 1.

### Sample analysis

TOF-SIMS analyses were performed with PHI-TRIFT-IV (ULVAC-PHI, Chigasaki, Japan), using a 60-keV Bi_3_^2+^ primary ion beam with a typical beam size of less than 0.4 μm^48^. The SIMS spectra were generated with a pulsed ion beam that had a duration of approximately 700 ps. Under computer control, the pulsed ion beam was rastered over the desired area to produce MS images. The mass analyzer of the PHI-TRIFT-IV is a triple focusing time-of-flight (TRIFT) mass analyzer based on three 90° electrostatic sectors and a pulse-counting secondary ion detector. The TRIFT analyzer records the mass spectrum at each X-Y pixel of the image generated by the rastered primary ion beam with a typical mass resolution > 15,000 M/Δm [full width at half maximum (FWHM)] and a mass range of over 10,000 *m/z*, where M is the *m/z* value of the peak of interest and Δm is the *m/z* full width at one half peak intensity of the peak of interest. The three electrostatic sectors in the TRIFT analyzer eliminate almost all metastable ions from the background in the mass spectra, which provided spectra with extremely high signal/background ratios for the organic samples used in this study. A pulsed low energy electron beam (~ < 10 eV) was used to provide charge neutralization for the insulating samples. The obtained mass spectra data for each X-Y point were processed and integrated using the WinCadenceN software (ULVAC-PHI) to visualize virtually the X-Y distribution of secondary ions of each *m/z* peak. Positive secondary ion spectra were obtained from 150 × 150 μm or 100 × 100 μm square areas. All the spectra were calibrated using C_2_H_5_, C_3_H_5_, and C_4_H_7_ peaks before data analysis. In low magnification analysis, TOF-SIMS images over a surface area of 2250 × 2250 μm were obtained at 256 × 256 pixel density after integration of 15 × 15 tiles, each having 256 × 256 pixel density, using WinCadenceN software (ULVAC-PHI). In high magnification analysis, approximately 5 × 10^7^ counts of secondary ions were obtained from a 100 × 100 μm square area, and images of each ion peak were generated at 256 × 256 pixel density. The representative images show typical regions chosen for the analysis in each case. To visualize the distribution of fluorescein via fluorescence microscopy, the samples were mounted in SCMM-R2 (Kawamoto's film method kit^49^; Leica Microsystems) and rapidly polymerized by UV irradiation to minimize delocalization of fluorescein. In some cases, the specimens were immunostained after TOF-SIMS analysis. Freeze-dried samples were immersed and blocked in phosphate-buffered saline (PBS) containing 10% fetal bovine serum (FBS) and 5% goat serum (Dako, Tokyo, Japan) for 30 min at room temperature and processed for immunostaining. The samples were incubated with primary antibodies in the blocking solution at 4°C for overnight, washed three times with PBS, and incubated with secondary antibodies in the blocking solution at room temperature for 1 h. Samples were washed with PBS, mounted in Mowiol (Merck, Darmstadt, Germany). The reagents and antibodies used were Hoechst 33342, Alexa 647-conjugated phalloidin (Invitrogen, Carlsbad, CA), anti-filaggrin (Covance, Berkeley, CA), anti-loricrin (Abcam, Cambridge, MA), anti-desmoplakin (DP-2.15 + DP-2.17 + DP-2.20 antibody cocktail; Progen, Heidelberg, Germany), anti-collagen type I (Abcam), and Alexa-conjugated secondary antibodies (Invitrogen).

### Superimposition *in silico*

The area analyzed by TOF-SIMS was subsequently analyzed by fluorescence microscopy. The area was imaged by laser confocal microscopy (TCS SP5; Leica Microsystems). TOF-SIMS images were superimposed on the immunofluorescent images using Photoshop CS4 software (Adobe, San Jose, CA). A methodological limitation exists to superimposing the images gained by two different methods. As the freeze-dried sample analyzed with TOF-SIMS was immersed in PBS for immunostaining and mounted with aqueous mounting medium, the sample swelled and became slightly deformed. Another challenge was that TOF-SIMS scans the surface of the samples, but confocal microscopy scans a cross section of the sample, which causes some positional distortions of the visualized structure depending on the *z*-axis. Thus, the two images were not completely superimposed.

### Reagents

Arginine, KCl, Cr(III)Cl_3_, K_2_Cr(VI)_2_O_7_, and fluorescein were purchased from Sigma-Aldrich (St. Louis, MO). Ceramide 6 was obtained from Cosmoferm BV (Delft, The Netherlands).

### Signal quantification and statistical analysis

Line scans were generated from the 100 × 100 μm acquired images using WinCadenceN software. The line scans across the image were positioned perpendicular to the imaged layers of interest. The line scan intensity of the desired m/z peaks is the average signal intensity based on a number of pixels parallel to any point along the line scan and within a symmetrical width of 50 μm. In the Cr permeation assay, the total counts of Cr ions detected from the 40 × 20 μm areas of the viable cell layers were compared. Multiple comparison tests were performed by Dunn's multiple comparison procedure using PRISM v6 software (GraphPad Software, La Jolla, CA).

## Author Contributions

Akiharu K. and Akiko K. designed the experiments. Akiharu K. performed the experiments and analyzed the data. I.I. operated the TOF-SIMS. H.K. provided filaggrin-deficient mice. Y.O. and K.N. helped with data analysis. M.A. supervised the project. Akiharu K. and M.A. wrote the manuscript.

## Supplementary Material

Supplementary Information Supplementary Information
