# Supplementary material for: The stratum corneum comprises three layers with distinct metal-ion barrier properties
Source: Sci Rep. 2013 Apr 25;3:1731. doi: 10.1038/srep01731 (PMC3635058; doi:10.1038/srep01731)
Supplement: Supplementary Information [file srep01731-s1.doc]

Supplementary Information

The stratum corneum comprises three layers with distinct metal-ion barrier properties

Akiharu Kubo^1,2,*^, Itsuko Ishizaki^3^, Akiko Kubo^4^, Hiroshi Kawasaki^1^, Keisuke Nagao^1^, Yoshiharu Ohashi^3^ and Masayuki Amagai^1^

^1^Department of Dermatology, Keio University School of Medicine, Tokyo 160-8582, Japan

^2^Center for Integrated Medical Research, Keio University School of Medicine, Tokyo 160-8582, Japan

^3^ULVAC-PHI INC., Chigasaki 253-0084, Japan

^4^Department of Biochemistry, Keio University School of Medicine, Tokyo 160-8582, Japan

Correspondence and requests for materials should be addressed to Akiharu Kubo (akiharu@a5.keio.jp)

The PDF file includes

**Supplementary Figure 1 |** Superimposition of the TOF-SIMS image of mouse skin on the immunofluorescence image.

**Supplementary Figure 2 |** Ion peaks of Na, K, and choline detected by TOF-SIMS.

**Supplementary Figure 3 |** Ion peaks of putative ceramide fragments detected by TOF-SIMS.

**Supplementary Figure 4 |** Ion peaks of arginine detected by TOF-SIMS.

**Supplementary Figure 5 |** Infiltration of Cr(IV) ions after soaking in aqueous 0.3 M K_2_Cr_2_O_7_ solution.

**Supplementary Figure 6 |** Ion peaks of Cr detected by TOF-SIMS.

**Supplementary Figure 7 |** Ion peaks of fluorescein detected by TOF-SIMS.

**Supplementary Figure 8 |** Infiltration of externally applied fluorescein, as visualized by TOF-SIMS and fluorescence microscopy.


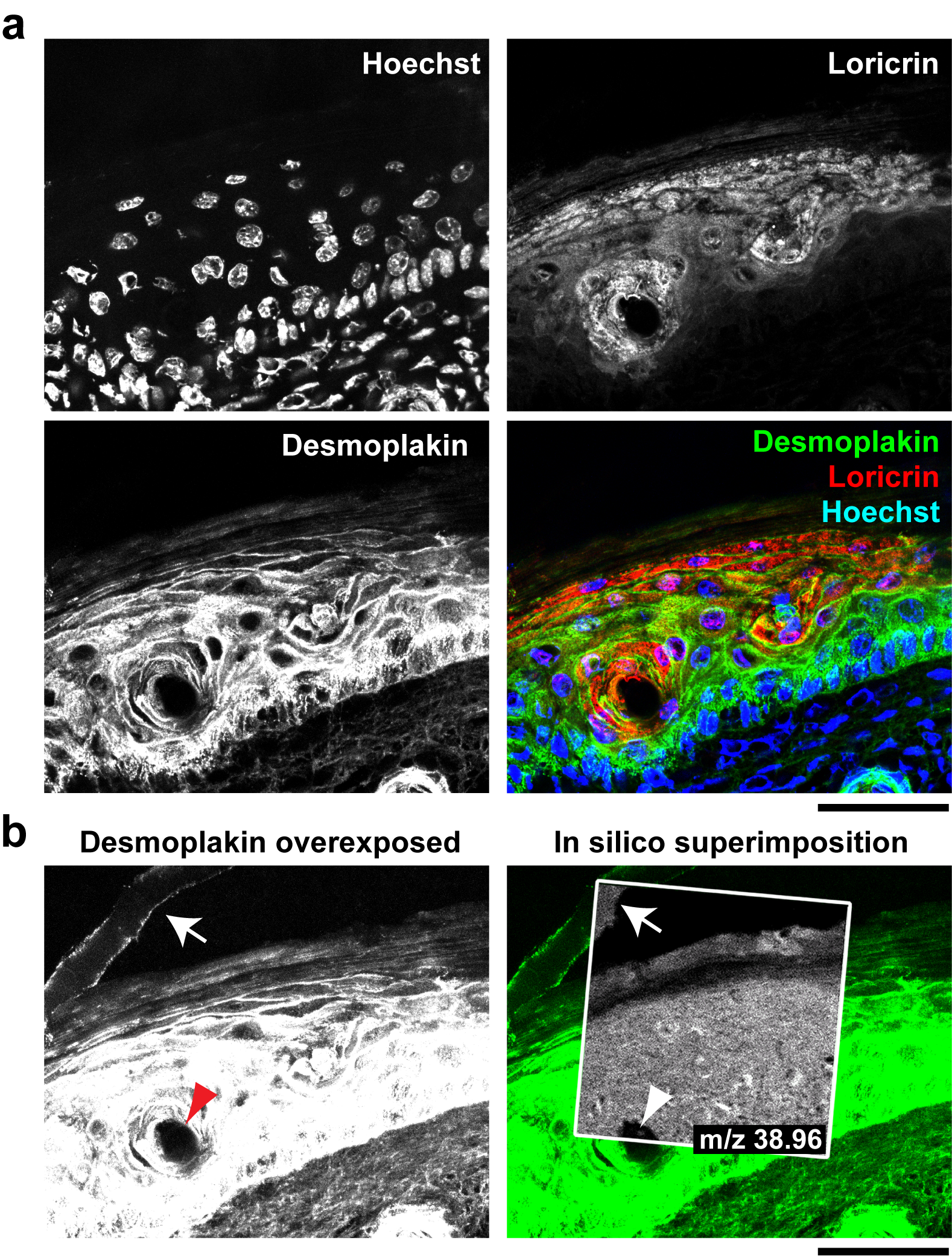
**Supplementary Figure 1 | Superimposition of the TOF-SIMS image of mouse skin on the immunofluorescence image.** (**a**) Immunofluorescence image of a skin section of mouse tail stained with Hoechst and anti-loricrin and anti-desmoplakin antibodies. (**b**) The center area of the immunofluorescence image had been analyzed by TOF-SIMS before immunostaining (the center square area of the right image). The overexposed image of desmoplakin staining (left panel and green in the right panel) and the *in silico* superimposed positive-ion micrograph of K both reveal a hair follicle (arrowheads) and desquamated cornified material (arrows), which were used as the position markers for superimposition. Positive-ion micrographs of the same area are presented in **Fig. 3b–d**. Scale bars, 50 μm. Each image is representative of three mice, for each of which two sections were investigated.


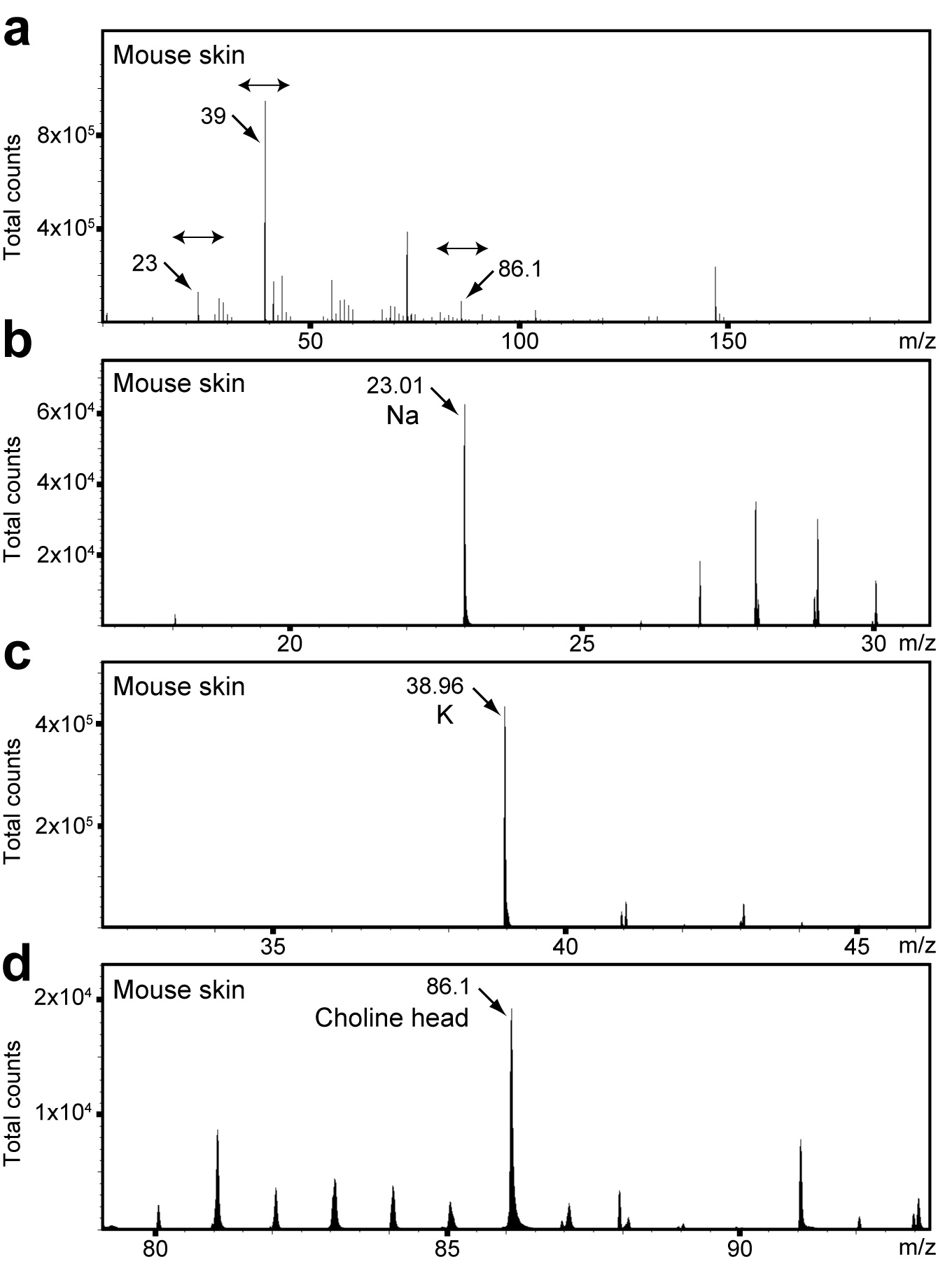


**Supplementary Figure 2 | Ion peaks of Na, K, and choline detected by TOF-SIMS.** Positive-mode TOF-SIMS mass spectra in the indicated m/z range from the flash-frozen, freeze-dried mouse tail section presented in **Fig. 3**. The ranges indicated by two-way arrows in (**a**) are shown in (**b**–**d**). The spatial distributions of the indicated ions (arrows) are presented in the TOF-SIMS images in **Fig. 3b**.


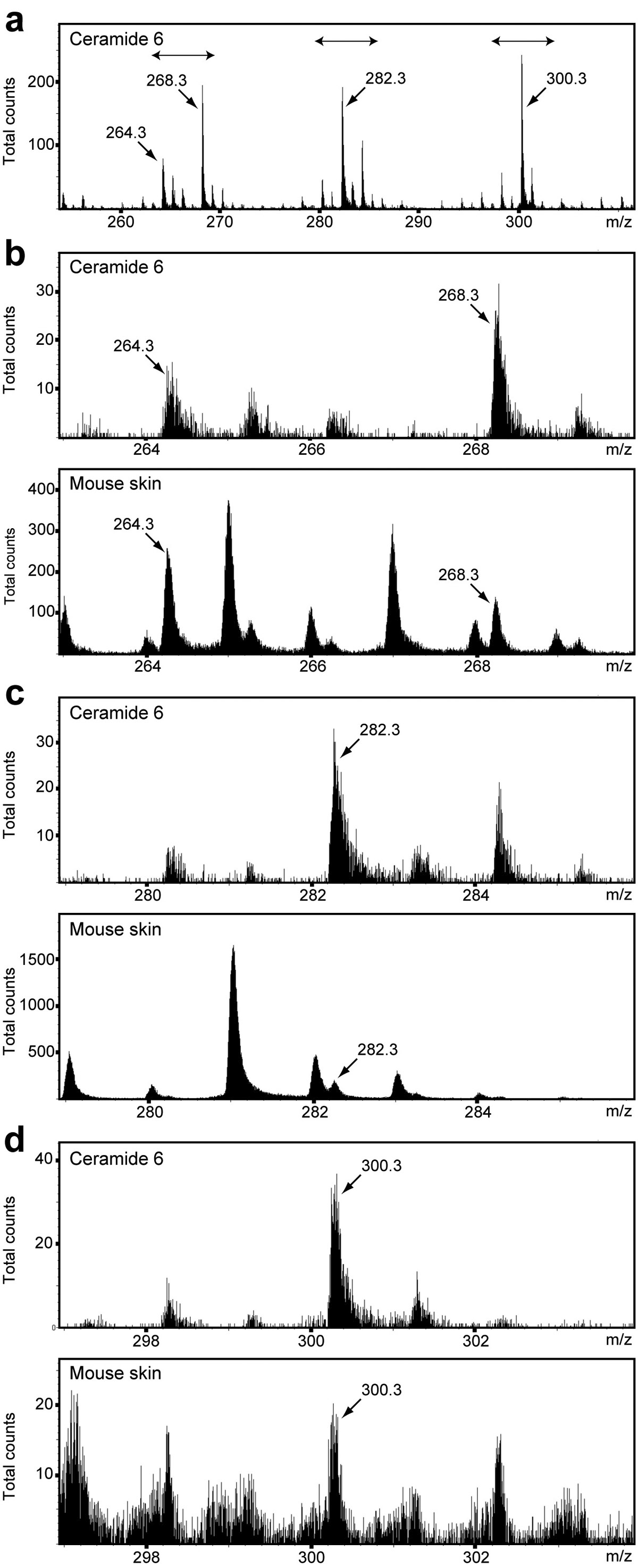


**Supplementary Figure 3 | Ion peaks of putative ceramide fragments detected by TOF-SIMS.** Positive-mode TOF-SIMS mass spectra in the indicated *m/z* range from purified ceramide 6 (**a** and upper panels of **b**–**d**) and from the mouse skin sections presented in **Fig. 3c** (**b**–**d**, lower panels). The ranges indicated by two-way arrows in (**a**) are shown in (**b**–**d**). The positive-mode mass spectra of purified ceramide 6 show four major peaks (arrows in **a**). Ion peaks with *m/z* of 264.3, 268.3, 282.3, and 300.3 were detected from skin sections (**b**–**d**, lower panels). These ions were specifically detected in the SC area of the skin, as shown in the TOF-SIMS images of these peaks presented in **Fig. 3c**.


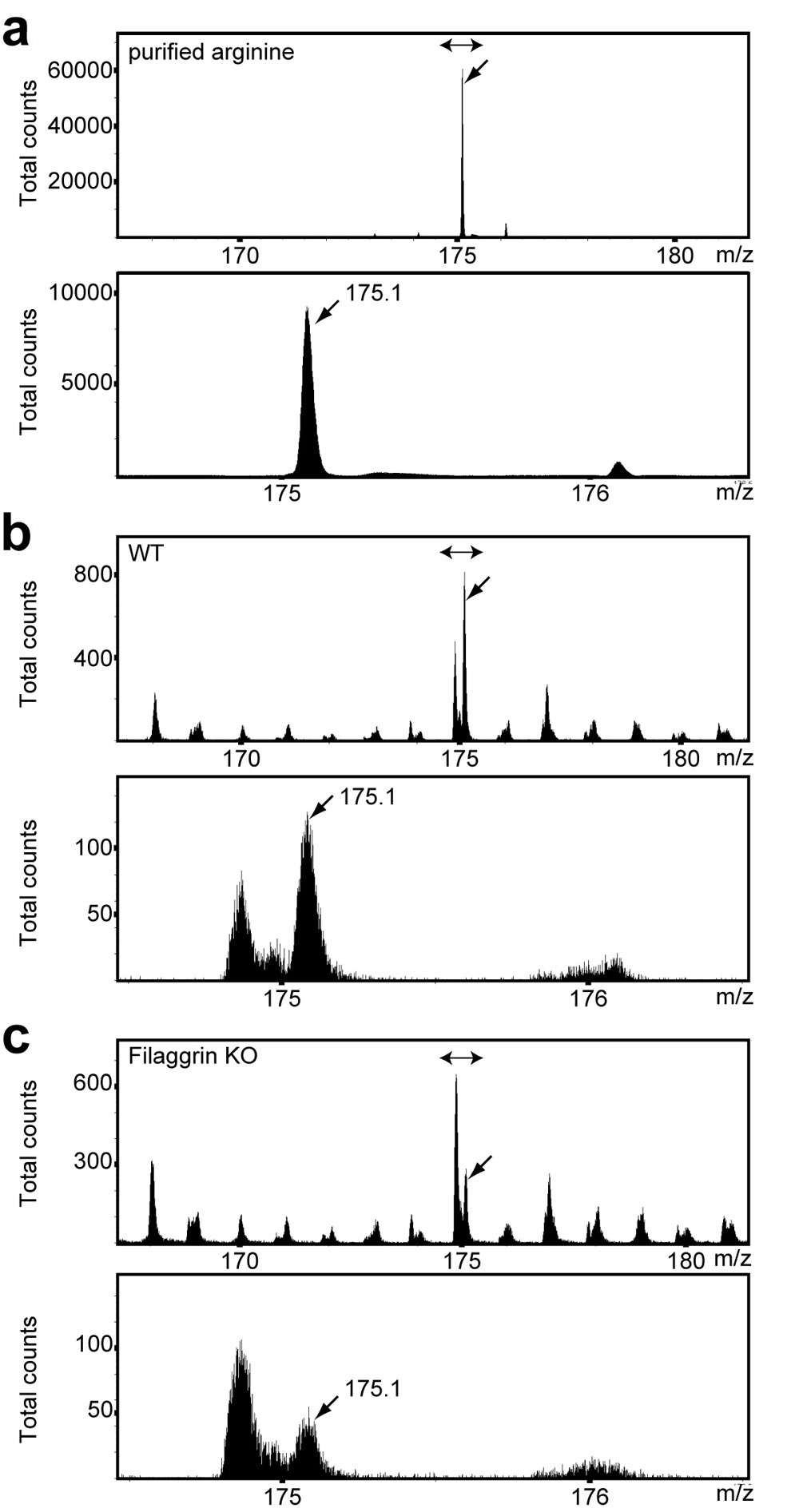


**Supplementary Figure 4 | Ion peaks of arginine detected by TOF-SIMS.** Positive-mode TOF-SIMS mass spectra in the indicated *m/z* range from purified arginine (**a**), from the skin section of the wild-type mouse presented in **Fig. 4a** (**b**), and from the skin section of the filaggrin-knockout mouse presented in **Fig. 4b** (**c**). The area indicated by a two-way arrow in each upper panel is enlarged in the respective lower panel. The major peak of arginine (*m/z* = 175.1) is indicated by arrows, and the TOF-SIMS image of this arginine peak is presented in **Fig. 4a and b**.


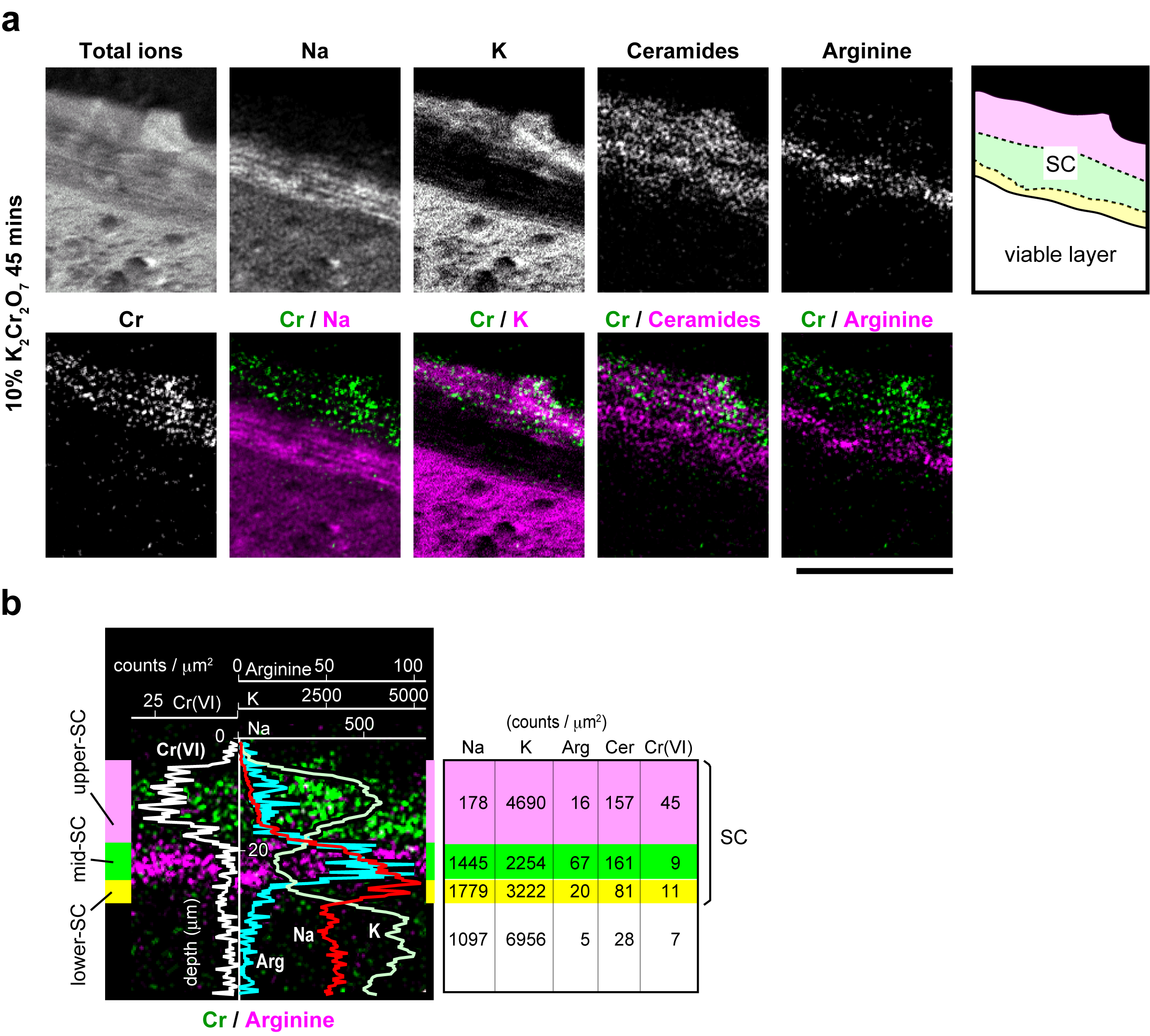
**Supplementary Figure 5 | Infiltration of Cr(IV) ions after soaking in 0.3 M K_2_Cr_2_O_7_ water solution.** (**a**) Representative positive-ion micrographs of skin sections after soaking in 0.3 M K_2_Cr_2_O_7_ water solution for 45 min. (**b**) The average line scan data for Na, K, arginine, and Cr(VI) for the area shown in (**a**) were overlaid on the image of Cr(VI)/arginine. The average signal count of each area is shown on the right. Each image is representative image of three mice, for each of which at least three sections were investigated.


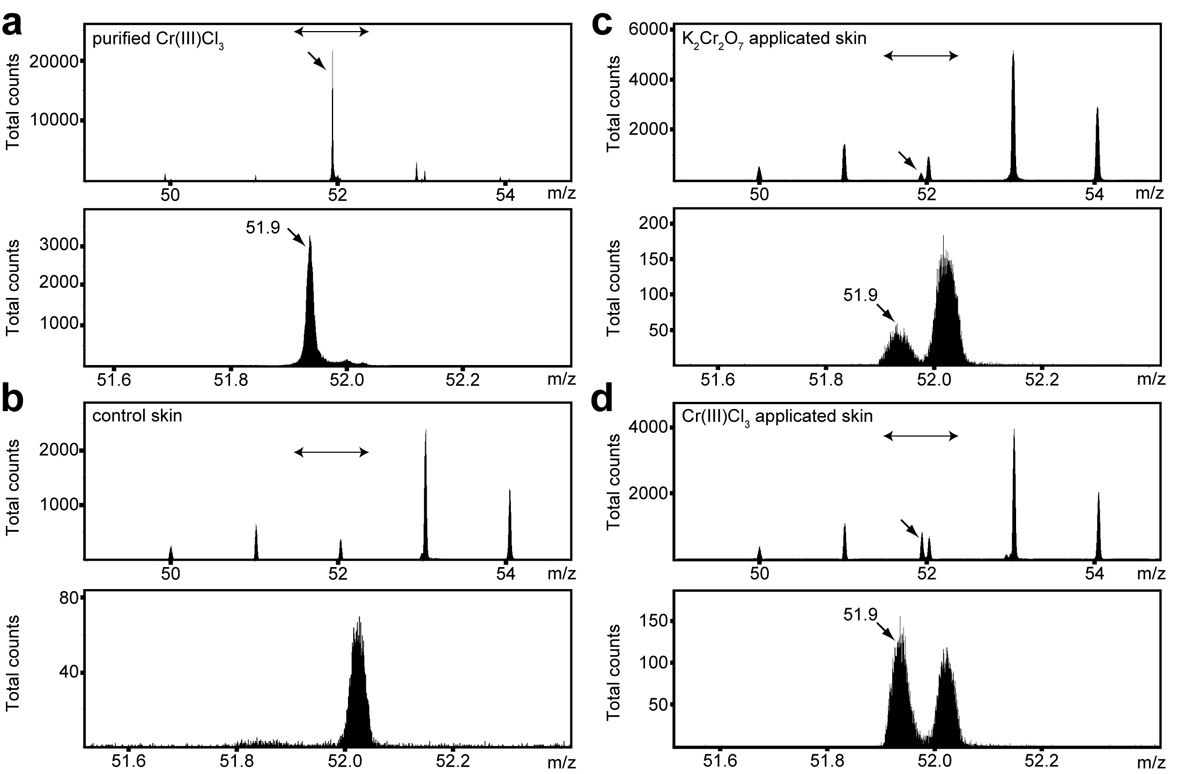


**Supplementary Figure 6 | Ion peaks of Cr detected by TOF-SIMS.** Positive-mode TOF-SIMS mass spectra in the indicated *m/z* range from purified Cr(III)Cl_3_ (**a**) and from a skin section from a control mouse (**b**) or skin sections from mice with K_2_Cr(VI)_2_O_7_ application (**c**) or Cr(III)Cl_3_ application (**d**). The area indicated by two-way arrows in each upper panel is enlarged in each respective lower panel. Specific peaks of Cr of *m/z* = 51.9 (arrows) were detected.


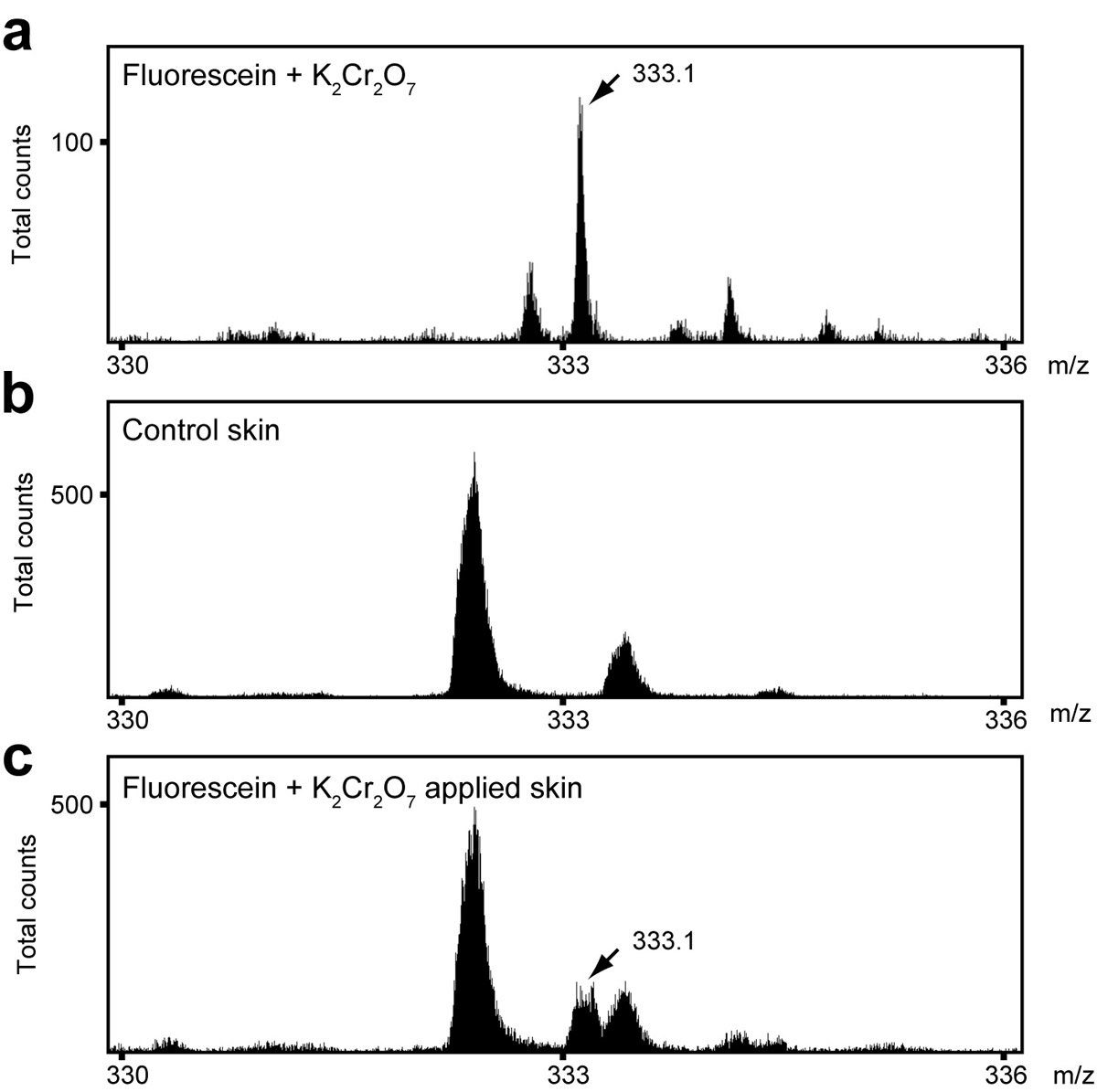


**Supplementary Figure 7 | Ion peaks of fluorescein detected by TOF-SIMS.** Positive-mode TOF-SIMS mass spectra in the indicated *m/z* range from fluorescein/K_2_Cr_2_O_7_ solution (**a**), from a skin section of a control mouse (**b**), and from a skin section of a fluorescein/K_2_Cr_2_O_7_-applied mouse (**c**). Specific peaks of fluorescein with *m/z* = 333.1 (arrows) were detected.


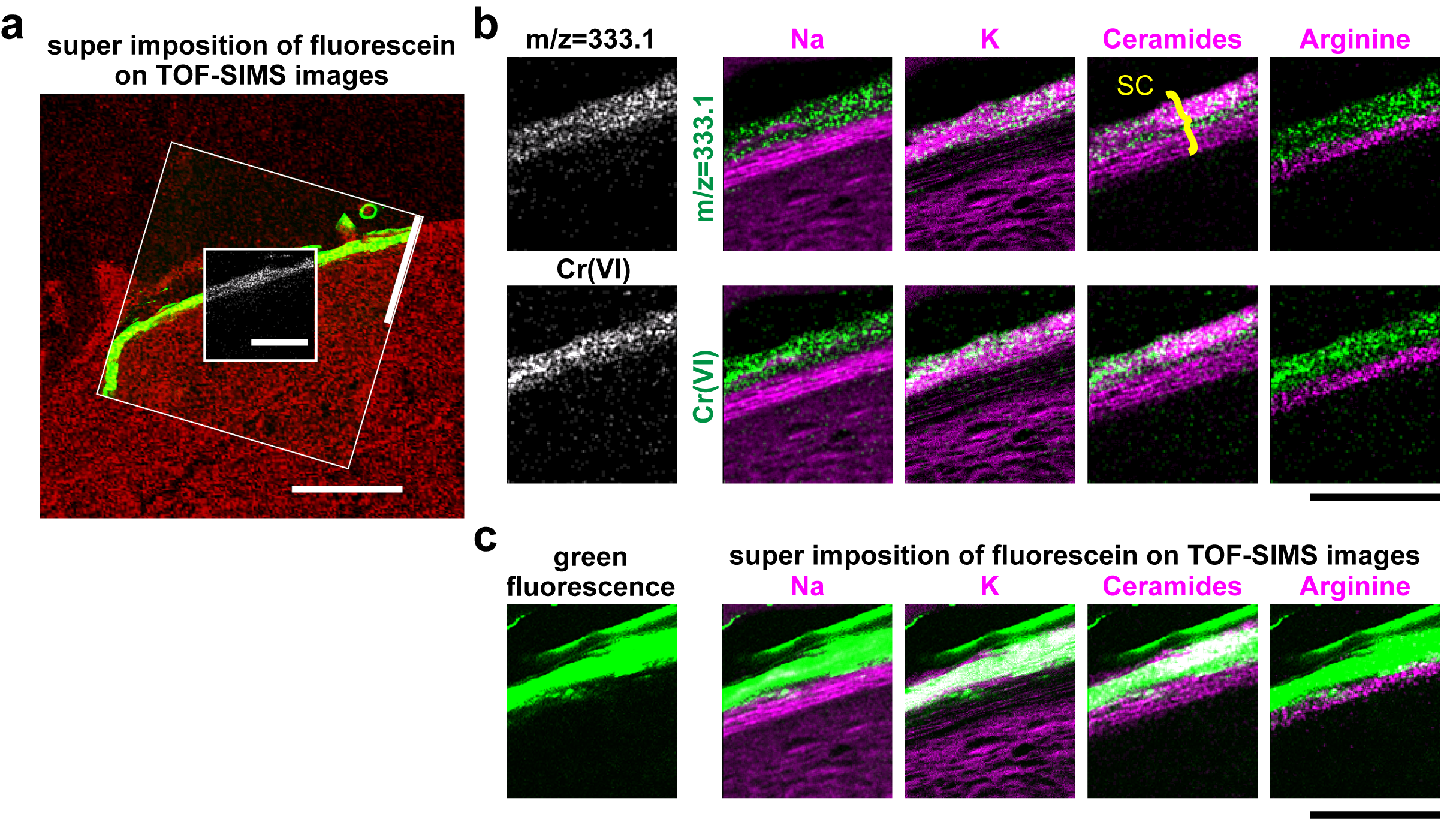


**Supplementary Figure 8 | Infiltration of externally applied fluorescein visualized by TOF-SIMS and fluorescent microscopy.** (**a**) Representative *in silico* superimposed image of positive-ion micrographs by TOF-SIMS on the green fluorescence image after soaking in 0.03 M fluorescein/0.3 M K_2_Cr_2_O_7_ solution. On top of the total positive-ion micrographs of skin sections (scale bar, 100 μm), a fluorescent image (dashed square; scale bar, 100 μm) and a high-resolution positive-ion micrograph of *m/z* = 333.1 (white-lined square; scale bar, 50 μm) were superimposed *in silico*. (**b**) Enlarged positive-ion micrographs of the center region shown in (**a**). TOF-SIMS images showing the distribution of fluorescein, observed as a peak of *m/z* = 333.1, and Cr(VI) were co-visualized in green with Na, K, ceramides, and arginine in purple. Scale bar, 50 μm. (**c**) *In silico* superimposition of green fluorescence on the TOF-SIMS images presented in (**b**). Scale bar, 50 μm. Each image is representative of three mice, for each of which two sections were investigated.
